# Risk factors for perinatal death in two different levels of care: a case–control study

**DOI:** 10.1186/1742-4755-11-11

**Published:** 2014-01-30

**Authors:** Paula Maria Silveira Soares Moura, Izildinha Maestá, Lígia Maria Souza Suppo Rugolo, Luís Felipe Ramos Berbel Angulski, Antônio Prates Caldeira, José Carlos Peraçoli, Marilza Vieira Cunha Rudge

**Affiliations:** 1 Postgraduation Program in Gynecology, Obstetrics and Mastology, Botucatu Medical School, São Paulo State University-UNESP, Botucatu, Brazil; 2 Department of Gynecology and Obstetrics, Botucatu Medical School, São Paulo State University-UNESP, Botucatu, Brazil; 3 Departament of Pediatrics, Botucatu Medical School, São Paulo State University-UNESP, Botucatu, Brazil; 4 Botucatu Medical School, São Paulo State University-UNESP, Scientific Initiation PIBIC/CNPq – Grant #º113024/2008-8, Botucatu, Brazil; 5 Departament of Women’s and Children’s Health, State University of Montes Claros-Unimontes (MG), Montes Claros, Brazil; 6 Department of Gynecology and Obstetrics, Botucatu Medical School, São Paulo State University-UNESP, Botucatu, Brazil

**Keywords:** Perinatal death, Risk factors, Care levels, Maternity

## Abstract

**Background:**

According to the World Health Organization, there are over 6.3 million perinatal deaths (PND) a year worldwide. Identifying the factors associated with PND is very helpful in building strategies to improve the care provided to mothers and their babies.

**Objective:**

To investigate the maternal, gestational and neonatal factors associated with PND at two different levels of care.

**Methods:**

Case–control study including 299 PND cases and 1161 infants that survived the early neonatal period (controls) between 2001–2006 in two hospitals at different care levels (secondary and tertiary) located in southeastern Brazil. Correlations between study variables and PND were evaluated by univariate analysis. PND-related variables were included in a multiple logistic regression model, and independent estimates of PND risk were obtained.

**Results:**

Although five-minute Apgar score <7, low birthweight and maternal hemorrhage were associated with PND in the secondary care center, no independent risk factors were identified at this level of care. In the tertiary hospital, PND was positively associated with primiparity, male sex, prematurity, low 5-minute Apgar score, and pregnancy complicated by arterial hypertension or intrauterine infection.

**Conclusions:**

Several risk factors positively associated with PND were indentified in the tertiary, but not in the secondary care level hospital. Since most of the risk factors herein identified are modifiable through effective antenatal and intrapartum care, greater attention should be given to preventive strategies.

## Introduction

Perinatal death (PND) is an important indicator of maternal care and of maternal health and nutrition. It also reflects the quality of obstetric and pediatric care available [1,2]. It has been associated to maternal, gestational and neonatal factors such as maternal age, parity, maternal diseases, multiple gestation, birth route and gestational age [2-4], as well as low birthweight and fetal growth restriction [2,4].

According to the World Health Organization (WHO) [5], there are over 6.3 million perinatal deaths a year worldwide. Of these, 2.64 million are stillbirths and 3.0 million are cases of early neonatal death. Ninety-eight per cent of these deaths take place in developing countries [6].

In Brazil, the few studies available on perinatal mortality focus on local realities and report rates two- to three-fold higher than those found in developed countries [1,3,7]. In the state of São Paulo, END rate was 15.2/1000 live births in 2005 [8]. In the municipality of Botucatu, the only records available cover the period of 1991/1992, when PND was 14/1000 livebirths [9].

Identifying the factors associated with PND is very helpful in building strategies to improve the care provided to mothers and their babies. Therefore, the objective of this study was to investigate the maternal, gestational and neonatal factors associated with PND at different care levels (secondary and tertiary) in Botucatu/SP, Brazil.

## Methods

In this case–control study, 299 PND cases and 1161 infants who survived the early neonatal period (controls) were assessed between January 1, 2001 and December 31, 2006 in two hospitals at different levels of care: Sorocabano Hospital (level II hospital), and Botucatu Medical School Hospital (level III-hospital), which are both located in Botucatu/SP, Brazil, and cover a population of approximately 1.2 million people. These regional maternal and perinatal services are essentially an institution-based hierarchical system with Level III hospital at the top. Care level was classified into two types based on the complexity of the treatments provided. Third level (higher complexity) hospital criteria included: complete maternity and neonatal care, intrapartum and neonatal intensive care, transport service, outreach education services, analysis and evaluation of new technologies. Second level (lower complexity) criteria included: 24-hour in-house anesthesia service, 24-hour clinical laboratory and radiology services, stabilization and transfer of complicated obstetric cases including preterm deliveries occurring before 34 weeks of gestation [10].

Cases were all stillbirths and early neonatal deaths documented during the study period in both participating hospitals. Infants who survived the early neonatal period were randomly selected to serve as controls. A 1:3 case to control ratio was used with week of birth being the matching criterion (α = 0.05, β = 0.20).

Data on maternal, gestational, and neonatal characteristics were obtained from hospital delivery and birth logs, medical records, necropsy reports, and death certificates. Maternal characteristics included age (< 20 years, 20–34 years, and ≥ 35 years), parity (primiparous or multiparous), and presence of hypertensive disorders, antepartum hemorrhage, hyperglycemia, intrauterine infection, urinary infection, and cardiovascular diseases. The same diagnostic criteria were followed in both participating hospitals (see box of definitions). Gestational characteristics consisted of gestation type (single or multiple), gestational age in weeks (determined by the mother’s last menstrual period or early ultrasound) stratified into seven groups (22–26, 27–28, 29–30, 31–33, 34–36, 37–41, 42–43 weeks), and delivery route (vaginal or Cesarean). Neonatal characteristics comprised newborn sex, one-minute Apgar score (≤3, 4–6 and ≥7), five-minute Apgar score (0–6 and ≥ 7), and birthweight in grams stratified in 6 groups (500-749 g, 750-999 g, 1000-1499 g, 1500-2499 g, 2500-3999 g, and ≥ 4000 g).

The study was approved by the Research Ethics Committee of Botucatu Medical School, São Paulo State University-Unesp (# 2234/2006).

### Definitions

Perinatal period: between 22 completed weeks (154 days) of gestation and six completed days after birth.

Perinatal death (PND): number of stillbirths (≥500 g or ≥22 weeks of gestation) and deaths in the first week of life per 1,000 live births [5].

Hypertensive disorders: gestational hypertension, preeclampsia, superimposed preeclampsia, and chronic hypertension [11].

Antepartum hemorrhage (APH): vaginal bleeding from 20–24 weeks of gestation to term, as a result of placental abruption or placenta previa

Hyperglycemia: normal 100-g GTT + abnormal GP or abnormal 100-g GTT + abnormal GP) [12].

Intrauterine infection: histological report (if available) indicating chorioamnionitis, definite or probable neonatal sepsis, or two or more of: maternal fever, maternal tachycardia, fetal tachycardia, uterine tenderness and increasing maternal leukocytosis) Urinary infection: > 100,000 organisms.

Data analysis was performed using SPSS/Windows® version 12 software. The associations between perinatal death and primiparity, hypertensive disorders, uterine infection, gestational age, sex, and Apgar score were assessed by calculating the odds ratio (OR) and 95% confidence intervals (95% CI) in a univariate analysis. Death-related variables were included in a multiple logistic regression model to obtain independent estimates of PND risk.

## Results

During the study period, 312 cases of PND occurred in the participating hospitals. Thirteen of these cases were excluded due to data unavailability. Of the 299 PND cases enrolled, 277 took place at the tertiary hospital while 22 occurred in the secondary hospital. A total of 1161 babies survived the early perinatal period (controls). Of these, 1081 were enrolled in the tertiary hospital (total births during the study period = 7470), and 80 in the secondary hospital (total births during the study period = 6699).

Tables 1, 2 and 3 show univariate analysis results regarding maternal, gestational, and neonatal factors associated with perinatal death in both study hospitals.

**Table 1:** Associations of perinatal death (PND) with maternal characteristics at both the tertiary and secondary care level hospitals (2001-2006)

**Maternal characteristic**	**Level III Hospital**				**Level II Hospital**			
	**PND**	**Controls**	**OR**	**p**	**PND**	**Controls**	**OR**	**p**
	**(n=277)**	**(n=1081)**	**(95% CI)**		**(n=22)**	**(n=80)**	**(95% CI)**	
**Maternal age (years)**								
20 -34 years	176 (63.5%)	682 (63.1%)	1.00		14 (63.6%)	51 (63.8%)	1.00	
< 20 years	73 (26.4%)	285 (26.4%)	0.99 (0.73-1.35)	0.969^(1)^	5 (22.7%)	21 (26.3%)	0.87 (0.28-2.71)	0.967^(1)^
≥ 35 years	28 (10.1%)	114 (10.5%)	0.96 (0.62-1.50)	0.863^(1)^	3 (13.6%)	8 (10.0%)	1.37 (0.32-5.84)	0.701^(2)^
**Parity**								
Multíparous	167 (60.3%)	811 (75%)	1.00		15 (68.2%)	59 (73.8%)	1.00	
Primíparous	110 (39.7%)	270 (25%)	1.97 (1.49-2.61)	< 0.001^(1)^	7 (31.8%)	21 (26.2%)	1.31 (0.47-3.65)	0.604^(1)^
**Hemorrhage**								
No	250 (90.2%)	1060 (98.1%)	1.00		19 (86.4%)	80 (100.0%)	1.00	
Yes	27 (9.8%)	21 (1.9%)	5.75 (3.17-10.42)	< 0.001^(1)^	3 (13.6%)	0 (0.0%)	*---*	0.009^(2)^
**Arterial hypertension**								
No	209 (75.5%)	930 (86.0%)	1.00		19 (86.4%)	78 (97.5%)	1.00	
Yes	68 (24.5%)	151 (14.0%)	2.00 (1.45-2.77)	< 0.001^(1)^	3 (13.6%)	2 (2.5%)	6.16 (0.96-39.48)	0.066^(2)^
**Intrauterine infection**								
No	230 (83.0%)	1047 (96.9%)	1.00		21 (95.5%)	78 (97.5%)	1.00	
Yes	47 (17.0%)	34 (3.1%)	6.49 (4.07-10.36)	< 0.001^(1)^	1 (4.5%)	2 (2.5%)	1.86 (0.16-21.49)	0.521^(2)^
**Diabetes**								
No	262 (94.6%)	1025 (94.8%)	1.00		22 (100.0%)	78 (97.5%)	1.00	
Yes	15 (5.4%)	36 (5.2%)	1.05 (0.58-1.88)	0.876^(1)^	0 (0.0%)	2 (2.5%)	*---*	1.000^(2)^
**Cardiopathy**								
No	269 (97.1%)	1059 (98.0%)	1.00		22 (100.0%)	79 (98.8%)	1.00	
Yes	8 (2.9%)	22 (2.0%)	1.43 (0.63-3.25)	0.389^(1)^	0 (0.0%)	1 (1.2%)	*---*	1.000^(2)^
**Urinary infection**								
No	239 (86.3%)	1045 (96.7%)	1.00		22 (100.0%)	79 (98.8%)	1.00	
Yes	38 (13.7%)	36 (3.3%)	4.62 (2.86-7.44)	< 0.001^(1)^	0 (0.0%)	1 (1.2%)	*---*	1.000^(2)^

^(1)^Chi-square test.

^(2)^Fisher’s exact test.

**Table 2:** Associations of perinatal death (PND) with gestational characteristics at both the tertiary and secondary care level hospitals (2001-2006)

**Gestational characteristics**	**Hospital level III**				**Hospital level II**			
	**PND**	**Controls**	**OR**	**p**	**PND**	**Controls**	**OR**	**p**
	**(n = 277)**	**(n = 1081)**	**(95% CI)**		**(n=22)**	**(n=80)**	**(95% CI)**	
**Gestation type**								
Single	236 (85.2%)	1023 (94.6%)	1.00		22 (100.0%)	78 (97.5%)	1.00	
Multiple	41 (14.8%)	(5.4%)	3.06 (2.00-4.68)	<0.001^(1)^	0 (0.0%)	(2.5%)	*---*	1.000^(2)^
**Gestational age (weeks)**								
37 - 41 (Term)	41 (14.8%)	644 (59.6%)	1.00		15 (66.6%)	68 (85.0%)	1.00	
42 - 43 (Post–term)	11 (0.4%)	65 (0.6%)	2.62 (0.31-21.80)	0.351^(2)^	1 (4.8%)	0 (0.0%)	*---*	0.180^(2)^
34 - 36 (Late pre–term)	40 (14.3%)	147 (13.6%)	3.92 (2.49-6.19)	<0.001^(1)^	0 (0.0%)	8 (10.0%)	*---*	0.348^(2)^
31 - 33 (Pre–term)	31 (11.2%)	69 (6.4%)	7.79 (4.61-13.16)	<0.001^(1)^	1 (4.8%)	1 (1.2%)	4.86 (0.29-82.38)	0.327^(2)^
29 - 30 (Extremely pre-term)	34 (12.3%)	17 (1.6%)	36.68 (18.93-71.09)	<0.001^(1)^	0 (0.0%)	0 (0.0%)	*---*	1.000^(2)^
27 - 28 (Extremely pre-term)	33 (11.9%)	19 (1.8%)	31.86 (16.70-60.78)	<0.001^(1)^	0 (0.0%)	2 (2.5%)	*---*	1.000^(2)^
24 - 26 (Periviable)	51 (18.5%)	86 (0.8%)	110.05 (50.83-238.28)	<0.001^(1)^	2 (9.5%)	1 (1.3%)	9.71 (0.82-114.67)	0.089^(2)^
22 - 23 (Not viable)	36 (13.0%)	32 (0.3%)	220.10 (65.04-744.79)	<0.001^(2)^	3 (14.3%)	0 (0.0%)	*---*	0.006^(2)^
**Type of delivery**								
Vaginal	171 (61.7%)	(54.9%)	1.00		17 (77.3%)	42 (52.5%)	1.00	
Cesarean	106 (38.3%)	(45.1%)	0.75 (0.57-0.98)	0.041^(1)^	5 (22.7%)	38 (47.5%)	0.32 (0.10-0.96)	0.037^(1)^

^(1)^Chi-square test.

^(2)^Fisher’s exact test.

**Table 3:** Associations of perinatal death (PND) with neonatal characteristics at both the tertiary and secondary care level hospitals (2001-2006)

**Neonatal characteristics**	**Hospital level III**				**Hospital level II**			
	**PND **^(*)^	**Controls**	**OR (95% CI)**	**p**	**PND **^(*)^	**Controls**	**OR (95% CI)**	**p**
	**(n = 130)**	**(n = 1081)**			**(n = 6)**	**(n = 80)**		
**Gender**								
Female	40 (30.8%)	491 (45.4%)	1.00		4 (66.7%)	34 (42.5%)	1.00	
Male	90 (69.2%)	590 (54.6%)	1.95 (1.32-2.88)	p < 0.001^(1)^	2 (33.3%)	46 (57.5%)	0.37 (0.06-2.14)	p = 0.398^(2)^
**1-minute Apgar score**								
≥ 7	84 (64.3%)	906 (83.8%)	1.00		2 (33.3%)	68 (85.0%)	1.00	
4 - 6	29 (22.2%)	109 (10.1%)	2.86 (1.78-4.59)	p < 0.001^(1)^	2 (33.3%)	9 (11.2%)	7.56 (0.43-131.63)	p = 0.238^(2)^
0 - 3	18 (13.5%)	66 (6.1%)	2.88 (1.61-5.15)	p < 0.001^(1)^	2 (33.3%)	3 (3.8%)	22.67 (1.12-456.81)	p = 0.107^(2)^
**5-minute Apgar score**								
≥ 7	56 (42.9%)	1071 (99.1%)	1.00		2 (33.3%)	80 (100.0%)	1.00	
0 - 6	74 (57.1%)	10 (0.9%)	141.87 (69.35-290.21)	p < 0.001^(1)^	4 (66.7%)	0 (0.0%)	*---* ( – )	p < 0.001^(2)^
**Birthweight (g)**								
2500 - 3999	23 (17.7%)	780 (72.2%)	1.00		4 (66.6%)	70 (87.5%)	1.00	
≥ 4000	0 (0.0%)	45 (4.2%)	*------*	p = 0.628^(2)^	0 (0.0%)	1 (1.3%)	*------*	p = 1.000^(2)^
1500 - 2499	29 (22.3%)	197 (18.2%)	4.97 (2.81-8.77)	p < 0.001^(1)^	1 (16.7%)	6 (7.4%)	2.92 (0.28-30.42)	p = 0.371^(2)^
1000 - 1499	13 (10.0%)	31 (2.9%)	14.22 (6.59-30.69)	p < 0.001^(1)^	0 (0.0%)	2(2.5%)	*---* ( – )	p = 1.000^(2)^
750 - 999	24 (18.5%)	14 (1.3%)	54.14 (26.68-126.66)	p < 0.001^(1)^	0 (0.0%)	0 (0.0%)	*---* ( – )	p = 1.000^(2)^
500 - 749	41 (31.5%)	13 (1.2%)	106.96 (50.57-226.22)	p < 0.001^(1)^	1 (16.7%)	1 (1.3%)	17.50 (0.92-334.13)	p = 0.128^(2)^

^(1)^Chi-square test; ^(2)^Fisher’s exact test;

(*)Deaths occurring within the first week of life (0-6 days).

Analysis of the associations between perinatal death and maternal characteristics revealed that increased death risk (p < 0.001, χ2 test) was associated with primiparity, hemorrhage, arterial hypertension, intrauterine infection and urinary infection in the tertiary level, whereas in the secondary level it was associated only with maternal hemorrhage (p = 0.009, Fisher’s exact test) (Table 1).

In relation to gestational characteristics, PND risk was three-fold increased by multiple gestations (OR = 3.06; CI 95% = 2.00 – 4.68; p < 0.001), and was inversely proportional to gestational age (p < 0.001 χ2 test or Fisher’s exact test) in the tertiary hospital. In the level II hospital, no significant association between gestational age and PND was observed (Table 2).

Of the 277 perinatal deaths occurring at the tertiary hospital, 130 (46.9%) occurred during the early neonatal period (from birth to 6 days of age). At this level of care, lower birthweight was associated with higher mortality (p < 0.001, χ2 test). Additionally, PND risk was increased two-fold in male infants (OR = 1.95; CI 95% = 1.32 – 2.88; p < 0.001), and a 1-minute Apgar score <7 led to a three-fold increase in the risk of PND (OR = 2.88; CI 95% = 1.61 – 5.15; p < 0.001), while a 5-minute Apgar score < 7 was associated with an increase of more than 140-fold in death risk (OR = 141.87; CI 95% = 69.35 – 290.21; p < 0.001) (Table 3). In the secondary hospital, few PND cases occurred during the early neonatal period (6/22; 27%), and only 5-minute Apgar score < 7 was associated with PND (p < 0.001, Fisher’s exact test) (Table 3).

Multivariate analysis showed that, at the level III hospital, primiparity, maternal hypertension, intrauterine infection, gestational age, male sex, and 5-minute Apgar score were significant risk factors for perinatal death (Table 4). In the level II hospital, no risk factors were significantly associated with PND.

**Table 4:** Multivariate analysis of the associations between independent variables and perinatal death at the tertiary care level hospital, 2001 - 2006

**Characteristic**	**β**	**p-value**	**OR**	**95% CI**
Interceptus	13.10	0.000	488618.38	
Primiparity	1.17	0.001	3.23	(1.62 – 6.43)
Arterial hypertension	0.98	0.010	2.65	(1.27 – 5.55)
Intrauterine infection	2.07	0.000	7.89	(2.66 – 23.39)
Gestational age (weeks)	-0.18	0.000	0.83	(0.78 – 0.89)
Male sex	0.84	0.024	2.31	(1.12 – 4.76)
5-minute Apgar score	-1.24	0.000	0.29	(0.23 – 0.37)

Deviance = 264.95; X^2^ = 539.05; df model = 6; p < 0.001.

OR = odds ratio.

## Discussion

Our results show that no maternal risk factor reached significance for PND at the level II hospital. This is likely to be due to the effectiveness of the referral program that incorporates the two hospitals participating in this study, which are identified according to the complexity of the treatment they provide. Thus, while all low-risk pregnant women were planned to deliver at the level II hospital, all high-risk pregnancies, including preterm deliveries (< 34 weeks of gestation), were referred to the level III hospital, unless they occurred urgently at the level II hospital before they could be referred [10].

In the participating level III hospital, primiparity, pregnancy complicated by arterial hypertension or intrauterine infection, prematurity, male sex, and low 5-minute Apgar score were found to be positively associated with perinatal death.

Other studies [13,14] have also described primiparity as an independent risk factor for PND. This might be due to the poor maternal adaptation to pregnancy caused by inadequate uterine vascular response to pregnancy hemodynamic demand. These abnormalities result in placental ischemia that leads to release of circulating angiogenic factors that cause preeclampsia. An ischemic intrauterine environment can also lead to intrauterine growth restriction and fetal distress [15].

In the secondary hospital, as maternal disease is a reason for referral to a tertiary center, the frequency of maternal diseases was low. In the tertiary hospital, however, there was a significant association between maternal diseases and perinatal mortality. The fact that arterial hypertension and intrauterine infection were major causes of PND in this care level underscores the impact of these conditions on the conceptus.

Studies conducted in Latin America [4,16], as well as in developed countries [17,18], have shown hypertensive disorders as the leading cause of PND. In pregnancies complicated by arterial hypertension, the impairment of uteroplacentary circulation may lead to intrauterine hypoxia or even stillbirth [19]. In addition, pregnancy-induced hypertension frequently causes prematurity, which also increases perinatal mortality.

Intrauterine infection increases the risk of neonatal death among both term and preterm infants [20,21]. Such complication may occur in the presence of prematurely ruptured ovular membranes with the ascent of cervico-vaginal bacteria into the uterus [20,21].

Twin pregnancy was associated with PND in univariate analysis, but it was not significant in the multivariate model. Multiple gestation poses high risks to both the fetuses and the mother [22], and is often associated with other risk factors such as low gestational age and low birthweight. In these cases, maternal and perinatal care should be provided at a tertiary care center [22,23]. It is worth of note that a multivariate logistic model was not attempted on the 22 PND cases in the level II hospital because of the small sample size.

Gestational age was an important risk factor for PND in the tertiary hospital. Indeed, a 17% decrease in PND risk was observed at each additional week of gestation. In the secondary hospital, preterm birth rate was low probably because preterm labour cases were referred to the tertiary hospital that has an Intensive Neonatal Care Unit.

The fact that prematurity rates are increasing worldwide, including Brazil [2,4,24], represents a major challenge to obstetricians and neonatologists seeking the reduction of perinatal mortality [24]. In developed countries, the threshold of viability at which the infant could have a 50% chance of survival if born prematurely is set at about 25 weeks of gestation [25,26]. In the participating tertiary hospital, the chance of death considerably dropped from 27 weeks of gestation onward, indicating a one-week reduction in fetal limit of viability as compared with local data from the past decade [27].

Although neonatal morbidity and mortality are primarily influenced by gestational age, birthweight (especially when < 750 g) can change infant outcome [26]. Almost one third of the babies that died in the participating tertiary hospital weighed less than 750 g. However, birthweight was not an independent risk factor for perinatal death, confirming that gestational age has a greater influence than birthweight on neonatal outcome.

Infant mortality rates are known to be higher among males than females, though the exact cause remains unclear [28,29]. In this study, male sex was associated with a two-fold increase in perinatal death chance in the tertiary hospital.

Low 5-minute Apgar score is one of the markers of perinatal asphyxia. In line with other reports [3,16,30], this study showed that 5-minute Apgar score <7 was a risk factor for PND. In a recent study of 19 Brazilian maternities and 15,169 liveborns, Pileggi et al. (2010) [31] demonstrated a significantly higher rate of early neonatal mortality among infants with 5-minute Apgar < 7 compared with infants with 5-minute Apgar ≥ 7 (25% vs. 0.3%, respectively). These data point to an important focus for intervention in order to improve perinatal outcomes: access to appropriate obstetric and neonatal care, particularly during labor and birth [3].

This study was limited by the small sample size of the level II hospital, and by its retrospective design, which did not allow considering all possible risk factors for perinatal mortality (e.g. congenital malformations, maternal smoking, syphilis, previous C-section, history of stillbirth, etc.) due to data unavailability. In spite of these limitations, our results show the importance of two-level referral systems for maternal and neonatal care based on the transport of high risk patients to the level of care offering the best chance for improved outcomes [10]. Nonetheless, as most of the risk factors herein identified may be modifiable through effective antenatal and intrapartum care, the adoption of preventive strategies should not be overlooked.

## Competing interests

The authors declare that they have no competing interests.

## Authors’ contributions

PMSSM and IM had the original idea for the study. LMSSR was responsible for the plan of analysis. PMSSM and LFRBA were responsible for data collection. All authors saw the output of analysis and tables and commented on their significance. PMSSM and IM wrote the first version of the manuscript, which was then reviewed, and corrected by APC, JCP and MVCR. All authors read the final version of the manuscript and agreed with its content before submission.
